# VviAGL11 self-regulates and targets hormone- and secondary metabolism-related genes during seed development

**DOI:** 10.1093/hr/uhac133

**Published:** 2022-06-10

**Authors:** Alessandra Amato, Maria Francesca Cardone, Nallatt Ocarez, Fiammetta Alagna, Benedetto Ruperti, Chiara Fattorini, Riccardo Velasco, Nilo Mejía, Sara Zenoni, Carlo Bergamini

**Affiliations:** Department of Biotechnology, University of Verona, 37134 Verona, Italy; Research Centre for Viticulture and Enology, Council for Agricultural Research and Economics (CREA), 70010 Turi, Italy; Instituto de Investigaciones Agropecuarias (INIA), Centro Regional de Investigación La Platina, Santiago RM 8831314, Chile; Trisaia Research Centre, National Agency for New Technologies, Energy and Sustainable Economic Development (ENEA), 75026 Rotondella, Italy; Department of Agronomy, Food, Natural resources, Animals and Environment, University of Padova, 35020 Padova, Italy; Department of Biotechnology, University of Verona, 37134 Verona, Italy; Research Centre for Viticulture and Enology, Council for Agricultural Research and Economics (CREA), 70010 Turi, Italy; Instituto de Investigaciones Agropecuarias (INIA), Centro Regional de Investigación La Platina, Santiago RM 8831314, Chile; Department of Biotechnology, University of Verona, 37134 Verona, Italy; Research Centre for Viticulture and Enology, Council for Agricultural Research and Economics (CREA), 70010 Turi, Italy

## Abstract

*VviAGL11,* the Arabidopsis *SEEDSTICK* homolog, has been proposed to have a causative role in grapevine stenospermocarpy. An association between a mutation in the coding sequence (CDS) and the seedless phenotype was reported, however, no working mechanisms have been demonstrated yet. We performed a deep investigation of the full *VviAGL11* gene sequence in a collection of grapevine varieties belonging to several seedlessness classes that revealed three different promoter-CDS combinations. By investigating the expression of the three *VviAGL11* alleles, and by evaluating their ability to activate the promoter region, we observed that VviAGL11 self-activates in a specific promoter-CDS combination manner.

Furthermore, by transcriptomic analyses on ovule and developing seeds in seeded and seedless varieties and co-expression approaches, candidate VviAGL11 targets were identified and further validated through luciferase assay and *in situ* hybridization. We demonstrated that *VviAGL11* Wild Type CDS activates *Methyl jasmonate esterase* and *Indole-3-acetate beta-glucosyltransferase*, both involved in hormone signaling and *Isoflavone reductase*, involved in secondary metabolism. The dominant-negative effect of the mutated CDS was also functionally ectopically validated in target induction. VviAGL11 was shown to co-localize with its targets in the outer seed coat integument, supporting its direct involvement in seed development, possibly by orchestrating the crosstalk among MeJA, auxin, and isoflavonoids synthesis.

In conclusion, the *VviAGL11* expression level depends on the promoter-CDS allelic combination, and this will likely affect its ability to activate important triggers of the seed coat development. The dominant-negative effect of the mutated VviAGL11 CDS on the target genes activation was molecularly validated. A new regulatory mechanism correlating *VviAGL11* haplotype assortment and seedlessness class in grapevine is proposed.

## Introduction

Seedlessness in table grapes is one of the most desired traits by consumers and for this reason, it is one of the most important targets of a breeding program. Seedlessness in grapes may arise from parthenocarpy or stenospermocarpy mechanisms: the former does not require pollination and the latter consists of an embryo abortion with consequent seed development regression after full fertilization [1]. Most seedless commercial table grapes are stenospermocarpic and the character is derived by crossings from the Thompson Seedless variety (other synonyms: Sultanina, Sultana) or their seedless descendants. In Thompson Seedless the first occurrence of stenospermocarpy took place in an unknown era: humankind selection and vegetative propagation preserved such an unfavorable trait and allowed it to spread. Over the last century, many studies tried to unravel the genetic mechanism behind stenospermocarpy in grapes, mostly by employing the pseudo-test cross F1 mapping population strategy [2]. Before the advent of molecular markers, which allowed the building of genetic maps, seedlessness was described as a quantitative character and so a model involving three loci regulated by a dominant locus was proposed [2, 3]. Costantini and colleagues identified the major QTLs on chromosome 18 and firstly proposed *VviAGL11* as a candidate gene for seedlessness in grapevine [1]. The nature of the dominant locus makes the character highly hereditable and perfectly suited for marker-assisted selection [4]. An efficient marker (named p3_VvAGL11) was further identified in the promoter region of the *VviAGL11* gene which proved the tight linkage between the seedlessness trait and this gene [5–7].

The *VviAGL11* is a member of the MADS-box transcription factor (TF) family reported for a broad range of species [8]. VviAGL11 has been described as homolog to SEEDSTICK (STK) or AGAMOUS-LIKE 11 (AGL11), which specifically controls ovule morphogenesis and seed coat differentiation in *Arabidopsis thaliana* [9].

Previous studies have demonstrated that *VviAGL11* has a causative role in stenospermocarpy in grape [5, 10]. Indeed, a full correlation was shown of a missense mutation Arg197Leu in exon 7 of *VviAGL11* with seedlessness in cultivated grapevine [11]. The mutated seedless version has a partial dominance over seeded alleles, allowing breeding for seedlessness based in heterozygous seedlings. Moreover, sequence data on the regulative region of *VviAGL11* showed low recombination rates between the promoter and the CDS regions, thus suggesting the existence of a Linkage disequilibrium in that region [12]. The existence of incomplete dominance of some alleles in the promoter region has been also observed [5, 10, 12]. Besides this genetic and transcriptional data confirming a major role of *VviAGL11* in grape stenospermocarpy, no functional evidence nor a working mechanism has yet been defined.

To this aim, we sequenced and analyzed the complete *VviAGL11* gene in grapevine varieties representing different seedlessness classes. We demonstrated the existence of specific promoter-coding sequence (CDS) combinations that directly affect the *VviAGL11* expression level. Transcriptomic analyses on ovule and developing seeds in seeded and seedless varieties highlighted the role of *VviAGL11* in hormone signaling and phenylpropanoid metabolism. In this regard, we identified and functionally validate a *Methyl jasmonate esterase*, an *Indole-3-acetate beta-glucosyltransferase,* and an *Isoflavone reductase*, as direct targets of *VviAGL11.* All our findings allowed us to define a regulatory mechanism correlating the haplotype assortment, the *VviAGL11* expression level, and seedlessness class in grapevine.

## Results

### VviAGL11 specific promoter-CDS combination

**Figure 1:**
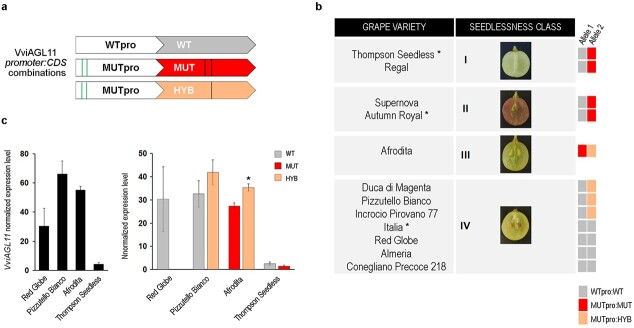
*VviAGL11* gene sequencing and expression analyses. **a** Schematic representation of the specific promoter-CDS association identified between the three main CDSs highlighted (WT, MUT, and HYB) and the promoters defined (WTpro and MUTpro). **b** Schematic representation of the specific *VviAGLL1* promoter-CDS combination identified in both alleles in seeded and seedless grape varieties. For varieties marked with an asterisk (^*^), the *VviAGL11* gene sequence was retrieved by previous works [13,14]. **c** Total *VviAGL11* expression level (on the left) and specific WT, MUT, and HYB *VviAGL11* CDSs expression level (on the right) in Red Globe, Pizzutello Bianco, Afrodita, and Thompson Seedless seeds at the pre-veraison stage. Quantitative real-time polymerase chain reaction (RT-qPCR) was performed as previously described [15]. Primer sets used are listed in Table S2 and primers allele specificity was demonstrated (Figure S1). Each value corresponds to the mean ± SD of three biological replicates relative to the *Glyceraldehyde-3-Phosphate Dehydrogenase-C2* internal control. Asterisks (^*^, p < 0.01) indicate significant diffrences. SD, Strandard Deviation.

The full gene sequence of *VviAGL11*, including the promoter region (−2213 to +6557 from transcription start site), was isolated and sequenced from nine grapevine varieties, representing different genetic backgrounds, and belonging to different seedlessness classes [6]. Red Globe and Almeria are seeded varieties, predicted as seeded genotypes by p3_VviAGL11 marker, while Supernova and Regal are representative of seedless varieties and predicted as seedless. Duca di Magenta, Pizzutello Bianco (named Incrocio Prosperi 105), and Incrocio Pirovano 77 are seeded varieties carrying the seedless allele defined by the p3_VvAGL11 marker. Conegliano Precoce 218 is a seeded variety characterized by the presence of a seedless-like variant for the p3_VvAGL11 marker. Finally, Afrodita is homozygous for the seedless allele of the p3_VviAGL11 marker and it is a stenospermocarpic cultivar with large green vital rudiments (Fig. 1).

Taking into consideration the already described *VviAGL11* gene sequence of the seeded Italia cultivar and the seedless Autumn Royal and Thompson Seedless cultivars [13, 14] three main CDSs were highlighted and named as follow: (i) seeded (wild type, WT), (ii) seedless (mutated, MUT) harboring the causative Arg197Leu substitution and the Thr210Ala substitution (iii) hybrid (HYB), sharing with the MUT variant only the Thr210Ala substitutions (Fig. 1a). The WT CDS was found in all analyzed cultivars except in Afrodita. The MUT CDS was found only in seedless varieties (i.e. Thompson Seedless, Regal, Supernova, Autumn Royal, and Afrodita) and HYB CDS was found in Afrodita, Duca di Magenta, Pizzutello Bianco, and Incrocio Pirovano 77 (Fig. 1b; Table S1). Concerning the regulatory region, many SNPs and INDELs were identified among different alleles. Based on sequence comparisons using accurate Sanger sequences from our recent study [12], we were able to define two main promoter types: (i) seeded (wild type promoter, WTpro) and seedless (mutated promoter, MUTpro). The main differences that distinguish promoters apparently reside in the INDELs that define the p3_VviAGL11 marker, indeed the largest sizes of such marker were clustered together in the MUTpro group (Figure S1). Interestingly, a full association between WTpro and WT CDS, and between MUTpro with either MUT or HYB CDS, was revealed, allowing us to define three specific promoter-CDS combinations (Fig. 1b).

The expression level of the WT, MUT, and HYB CDSs was assessed in four varieties representing different promoter-CDS combinations: Red Globe was analyzed as homozygous for the WT allele, Pizzutello Bianco as heterozygous with WT and HYB alleles, Thompson Seedless as heterozygous with WT and MUT alleles, and Afrodita as heterozygous cultivar with HYB and MUT alleles. The analysis was performed on developing seeds collected at the pre-veraison stage (E-L stage 34) at which *VviAGL11* reaches a high level of expression [5]. The highest total *VviAGL11* expression was found in Pizzutello Bianco, with a slightly higher expression level of HYB allele (p < 0.005) (Fig. 1c). In Red Globe, the total expression of *VviAGL11* was about half the expression observed in Pizzutello Bianco, although a very similar expression level of WT allele was detected in the two seeded varieties. In Thompson Seedless, we reported the lowest *VviAGL11* total expression level, with a slightly higher expression of WT allele although not statistically significant. Noteworthy, in Afrodita, a stenospermocarpic variety that produces vital, large but not lignified large rudiments, the total expression of *VviAGL11* resulted very high, with the HYB allele more expressed than MUT (p < 0.01).

### VviAGL11 regulates its transcription in a specific promoter-CDS manner

**Figure 2:**
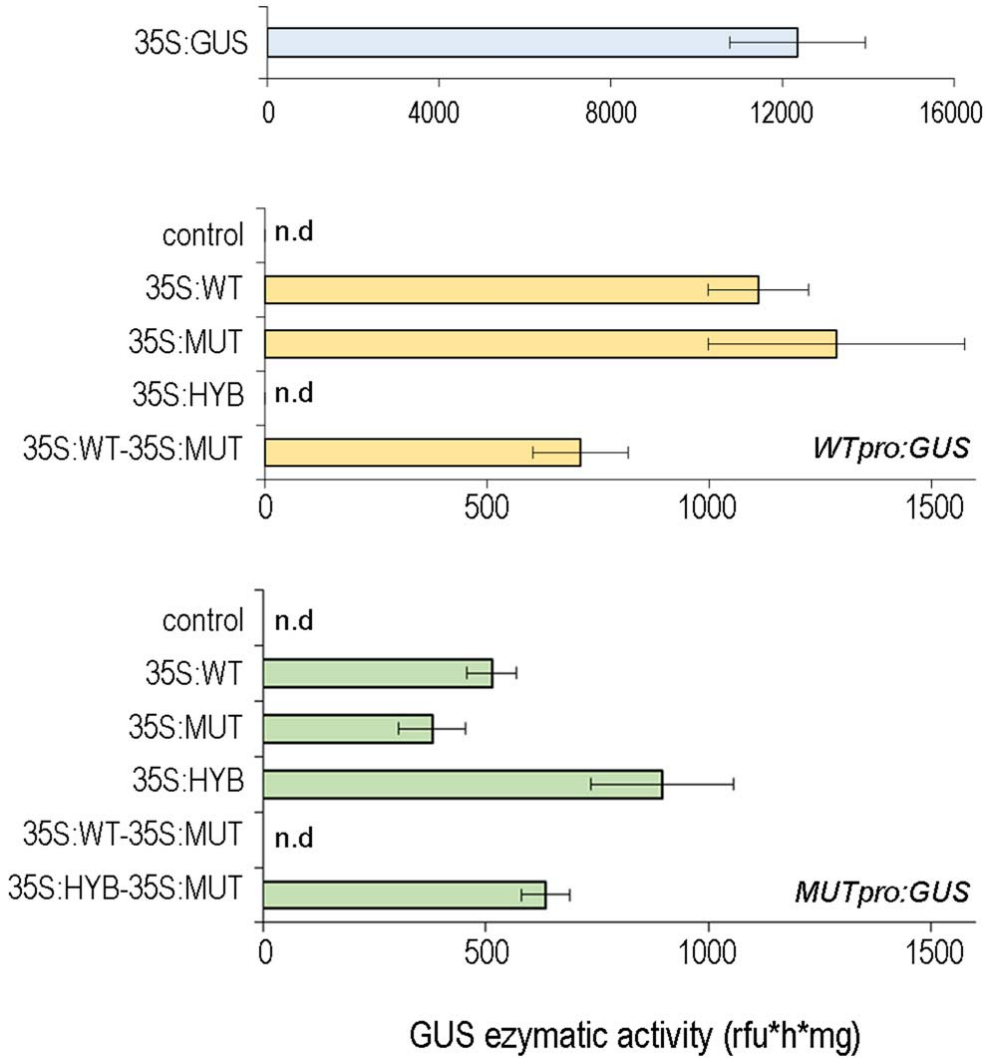
VviAGL11 self-activation. The ability of WT, MUT, and HYB CDSs to activate both WT and MUT VviAGL11 regulative regions was assessed by performing a GUS fluorimetric assay in Nicotiana benthamiana leaves. Plants agroinfected with a 35S:GUS construct as positive control shows a strong GUS enzymatic activity, while no activity was detected in negative controls performed by infiltrating plants with only reporter vectors (WTpro:GUS or MUTpro:GUS constructs). Each test was performed in biological triplicate and each value was measured in triplicate. Each value corresponds to the mean ± SE. SE, Standard error.

Different combinations of the three CDSs and WT and MUT promoter were tested by GUS fluorimetric assay in agroinfiltrated *Nicotiana benthamiana* leaves demonstrating that VviAGL11 can activate its own transcription (Fig. 2). Both WT and MUT CDSs strongly activate *WTpro*, to a similar extent, whereas HYB CDS is completely unable to activate it. The combined action of WT and MUT CDSs can still activate *WTpro* but to a considerably lesser extent. On the other hand, all CDSs activate *MUTpro*, with a significantly stronger induction (p < 0.05) exerted by HYB CDS in comparison with WT and MUT (Fig. 2). Moreover, when MUT CDS is used together with HYB CDS, the MUTpro is still induced, supporting the *VviAGL11* expression pattern found in Afrodita (Figure 1c). Nevertheless, MUT CDS partially inhibits the ability of WT and HYB to activate WT and MUT regulative region respectively.

Interestingly, *MUTpro* activation by WT and MUT CDSs resulted significantly lower (p < 0.05) than that observed for *WTpro*, suggesting that the proposed VviAGL11 self-activation mechanism depends on the specific promoter sequence rather than Arg197Leu substitution in the CDS. In line with this hypothesis, we observed that when both WT and MUT CDSs are simultaneously used, no activation was detected for *MUTpro* (Fig. 2).

Moreover, the HYB alleles showed an opposite behavior of WT and MUT CDSs, being able to strongly activate *MUTpro* and not *WTpro*. This could suggest a peculiar mechanism of *VviAGL11* expression induction exerted by HYB CDS.

Altogether these results suggest that VviAGL11 regulates its own transcription.

By searching for *cis*-elements in the WT VviAGL11 regulative region, we found four CArG boxes (consensus sequence “CC[A/T]_6-8_GG”; Figure S3a). CArG boxes have been described as MADS proteins binding site [16,17] and we can therefore suppose a physical interaction between VviAGL11 protein and VviAGL11 promoter. Moreover, a preliminary protein-DNA affinity assay based on DAP-seq assay supports this hypothesized interaction and suggests that all CArG boxes identified could be required and deserve further investigation (Figure S3b).

### Seeded and seedless varieties feature different gene modulation during ovule and seed development

**Figure 3:**
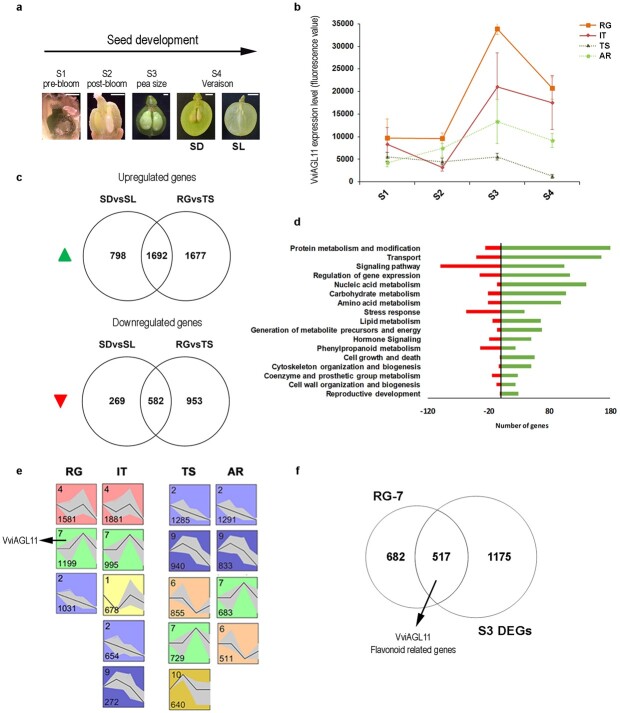
Gene modulation during ovule and seed development. **a** Images of ovules and seeds sampled at four developing stages in seeded (SD) and seedless (SL) varieties from pre-bloom to veraison. S1, pre-bloom, E-L 17; S2, post-bloom, E-L 19–25; S3, pea size, E-L 29; S4, veraison, E-L 35. **b** The VviAGL11 expression level in each variety at each developing stage was extracted from the entire transcriptomic data (Data S1). **c** Venn diagrams showing genes up and downregulated (p < 0.05; |FC| > 2) between seeded (Italia and Red Globe) and seedless (Thomson seedless and Autumn Royal) varieties (SDvsSL), and those between Red Globe and Thompson Seedless varieties (RGvsTS) at seed developing stage S3. **d** Functional categories distribution of common differentially expressed genes (DEGs) between two transcriptomic subsets (SDvsSL and RGvsTS). Only categories including ≥20 up or downregulated genes are shown. **e** Significant profiles (<5% Bonferroni correction method) of 5971 DEGs identified at S3 stage (SDvsSL and/or RGvsTS) during the entire ovule and seed development, among 15 profiles subjected to STEM analysis, in seeded and seedless varieties. In each frame, the number of genes is displayed bottom left, the ID number is shown top left, gray curves represent individual profiles, and the black line represents average profile. The x-axis represents sampling points, and y-axis denotes log2 scale fold change in expression value. **f** Venn diagrams showing genes clustering with VviAGL11 in Red Globe (RG-7; 1199) and those upregulated (p < 0.05; FC > 2) in both SDvsSL and RGvsTS comparison at stage S3 (S3 DEGs; 1692). RG, Red Globe; IT, Italia; TS, Thompson Seedless; AR, Autumn Royal.

A transcriptomic assay on ovules and seeds collected at four developmental stages (Fig. 3a; S1, pre-bloom, E-L 17; S2, post-bloom, E-L 19–25; S3, pea size, E-L 29; S4, veraison, E-L 35) was conducted in two seeded (SD), and two seedless (SL) varieties (Data S1).

We sampled ovule and seeds from pre-bloom to veraison, which is the time interval in which the seed abortion occurs in stenospermocarpic varieties. The two seeded varieties, Red Globe (RG) and Italia (IT), are the most diffused and commercially important table grape cultivars, in particular appreciated for their berry size. Regarding the two seedless varieties, Autumn Royal (AR) and Thompson Seedless (TS), AR is interesting to study as it is a stenospermocarpy cultivar of class II featuring small rudiments, and TS is the cultivar in which the mutation leading to stenospermocarpy first appears. *VviAGL11* showed a similar expression profile in SD varieties during seed development*,* with a flat low expression at S1 and S2 stages, a sharp increase at S3, and a decrease at S4. In TS, *VviAGL11* expression was very low throughout seed development and the peak of expression at S3 stage is no longer recognized. AR presented an intermediate expression trend, with a peak of expression at S3, but a lower average expression in comparison to SD varieties (Fig. 3b).

Differentially expressed genes (DEGs) between SD and SL varieties at each developmental stage, were then investigated. We identified 640 DEGs (p < 0.05; |FC| > 2) at S1, 1297 at S2, 3341 at S3 and 1777 at S4 (Data S2, Figure S4). The highest number of DEGs identified at the S3 stage together with the highest percentage of genes specifically modulated in S3, support the hypothesis of a VviAGL11 key role in seed formation as the highest difference of *VviAGL11* expression level between SD and SL varieties was observed at the same stage**.**

Thus, to better focus on those genes likely linked to seedlessness by a putative VviAGL11-related mechanism, we also identified DEGs at the S3 stage between Red Globe and Thompson Seedless (RGvsTS) that in accordance with phenotypic differences are the seeded and seedless varieties with the higher and lower VviAGL11 expression levels, respectively (Figure 3b). In the RGvsTS comparison at stage S3, we identified 4904 DEGs (p < 0.05; |FC| > 2) among which 3369 upregulated and 1535 downregulated in RG with respect to TS (Data S3). The comparison of these genes with the 2490 upregulated and the 851 downregulated DEGs obtained in SDvsSL comparison at S3 stage, revealed 1692 commonly up-, and 582 commonly down-regulated genes that we grouped according to their functional category (Fig. 3c and d; Data S4). Genes commonly upregulated proved to be mainly involved in “Protein metabolism and modification”, “Nucleic acid metabolism” and in “Transport”. Also, several genes belonging to “Regulation of gene expression” category are significantly upregulated in SD varieties in comparison to SL ones, such as 4 MADS-box family transcription factors among which the *VviAGL11*, the *inner no outer* gene that is a member of C2C2-YABBY family transcription factor involved in growth of the outermost cell layer of integuments (Skinner, Brown et al. 2016), the *endosperm defective 1* (EDE1), the *embryo defective 1674* (*EMB1674*), 11 bHLH transcription factors among which the transparent testa *VviMYC1*, 9 MYB family transcription factors, and 4 Squamosa promoter-binding proteins (Data S4). We also highlighted that the “Cell growth and death” category, including 12 cyclins and several cellular division proteins, and “Reproductive development” category, are specifically represented by upregulated DEGs (Fig. 3d). Moreover, although genes involved in “Hormone signaling” were found among both up and down DEGs, we observed that genes related to Auxin metabolism, such as an *indole-3-acetate beta-glucosyltransferase*, two *IAAs*, two *ARFs,* and *PIN1*, are mainly induced in SD varieties (Data S4).

Conversely, no functional categories were peculiarly represented by the commonly downregulated genes, which are mainly involved in “Signaling pathway” and “Stress response” (Fig. 3d; Data S4).

We finally looked at the expression profile of all 5971 DEGs identified at S3 stage (SDvsSL and/or RGvsTS) during the entire ovule and seed development (Data S1). By applying a Short Time-series Expression Miner (STEM) analysis to the four variety datasets, distinct profile sets between SD and SL varieties were identified (Figure S5, Data S5). Focusing on significant patterns, we noted that gene clusterization is almost conserved between RG (cluster 4,7,2) and IT (cluster 4,7,12, 9) while different profile sets feature TS (cluster 2,9,6,7,10) and AR (cluster 2,9,7,6) with the most abundant clusters showing a decreasing expression profile (Fig. 3e). Moreover, a deep inspection of gene expression clusterization revealed that in seeded varieties *VviAGL11* belongs to similar clusters (7 and 5) whereas in TS and AR it was not (cluster 4 and 14 in TS and AR respectively). Interestingly, a cluster with an opposite expression profile to the one of VviAGL11 (cluster 6) resulted among significant profiles in seedless varieties (Figure S5).

Among the *VviAGL11-*including clusters, only the RG-7 cluster resulted significant and it included the highest number of genes (1199) in comparison to the others. By inspecting the 1199 RG-7 genes, we observed that a large amount of them (517) are shared with the 1692 commonly upregulated DEGs at S3 stage (S3 DEGs) (Fig. 3f). Although the functional category distribution of those 517 genes is largely consistent with what is reported in Fig. 3d (i.e “Protein metabolism and modification”, “Nucleic acid metabolism” and “Transport” are widely represented), we observed that many genes are involved in “Phenylpropanoid metabolism”. Interestingly, among DEGs belonging to the phenylpropanoid pathway, 6 *isoflavone reductases* were identified and all of them were upregulated in SD varieties and clusterized with *VviAGL11* in the RG-7 cluster.

### Identification of candidate VviAGL11 target genes

**Figure 4:**
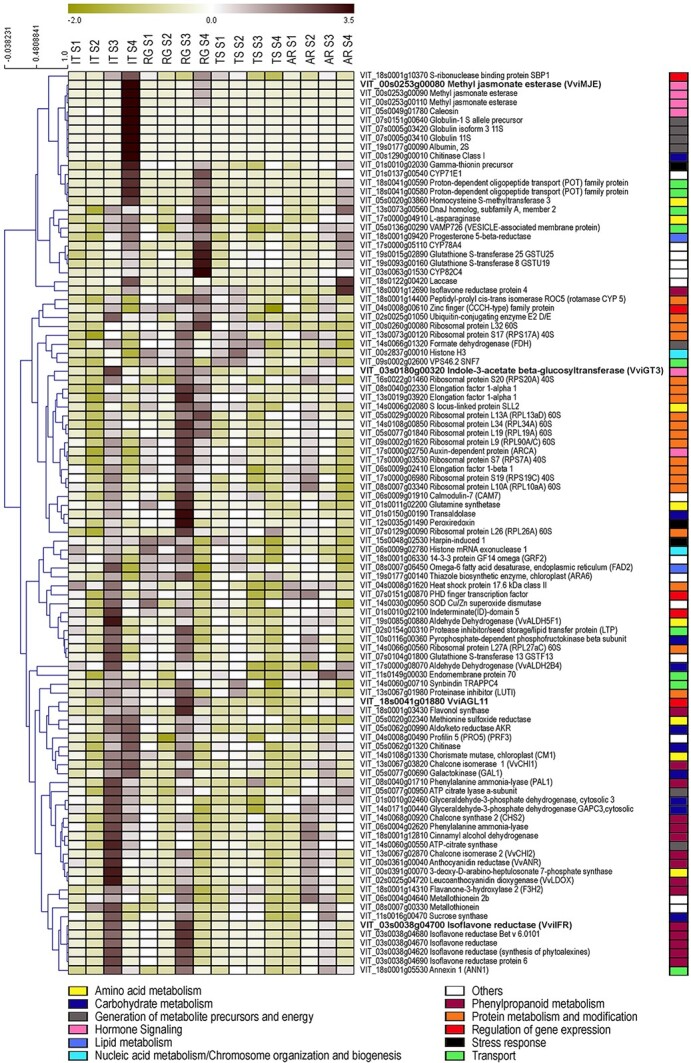
Expression pattern of VviAGL11 and the top 100 VviAGL11 co-expressed genes in the entire ovule and developing seed transcriptome. The heat map and hierarchical clustering (HCL) analysis were performed by using TMeV software (https://sourceforge.net/projects/mev-tm4/). Functional categories of investigated genes are reported according to the legend. “Others” category includes those categories with a maximum of two genes (i.e Cellular homeostasis, Coenzyme, and prosthetic group metabolism, Cytoskeleton organization and biogenesis, Organelle organization and biogenesis, Oxidation–reduction, Terpenoid metabolism, Signaling pathway). VviAGL11 and its candidate target genes (VviMJE, VviGT3, and VviIFR) are reported in bold.

To identify putative target genes of VviAGL11 we performed a multi-*VviAGL11* co-expression analysis (Data S6). We investigated genes that were highly positively and negatively co-expressed with *VviAGL11* in the entire developing ovule and seeds from Data S1, in the grapevine Corvina expression atlas [18] and VTC database [19], taking into consideration that VviAGL11 may act as both activator and repressor. Genes co-expressed with *VviAGL11* in at least one dataset were listed (n = 6967 in Data S6). In order to identify the most confident putative VviAGL11 targets among co-expressed genes, we considered other parameters, such as the intensity of correlation with *VviAGL11* given by multiplying each value of correlation from different data sources, the down-regulation behavior in seedless in comparison to seeded varieties during three stages of seed development reported in a previous study (Wang, Hu et al. 2016), and the average expression level in ovules and seed samples (from Data S1). The final score of each gene, given by considering all the above-described criteria, defined a top-100 list of candidates VviAGL11 target genes (Fig. 4).

Several of the top-100 genes belonged to the primary metabolism category (i.e protein and carbohydrate metabolism), according to functional category distribution of upregulated DEGs at S3 in SD vs SL varieties (Fig. 3d). Interestingly, genes involved in the flavonoid pathway and hormone signaling were also found (Data S6). Among genes belonging to these categories, we identified a *Methyl jasmonate esterase* (*VviMJE*; *VIT_00s0253g00080*), an *Indole-3-acetate beta-glucosyltransferase* (*VviGT3*; *VIT_03s0180g00320*), and an *Isoflavone reductase* (*VviIFR*; *VIT_03s0038g04700*), at the top of the ranking.

Notably, these genes resulted differentially expressed in S3 in both SDvsSL and RGvsTS comparisons. Moreover, by looking at their clusterization, obtained by STEM analysis, we observed that *VviMJE* clusterized with *VviAGL11* in Autumn Royal (AR-14), *VviGT3* belonged to the Red Globe cluster 5 (RG-5) that is very close to the RG-7 *VviAGL11*-including cluster, and *VviIFR* clusterized with *VviAGL11* in the RG-7 cluster (Data S5). We, therefore, selected these three genes as ideal VviAGL11 candidate targets for further experimental investigations.

### VviAGL11 controls the expression of *VviMJE*, *VviGT3* and *VviIFR*

**Figure 5:**
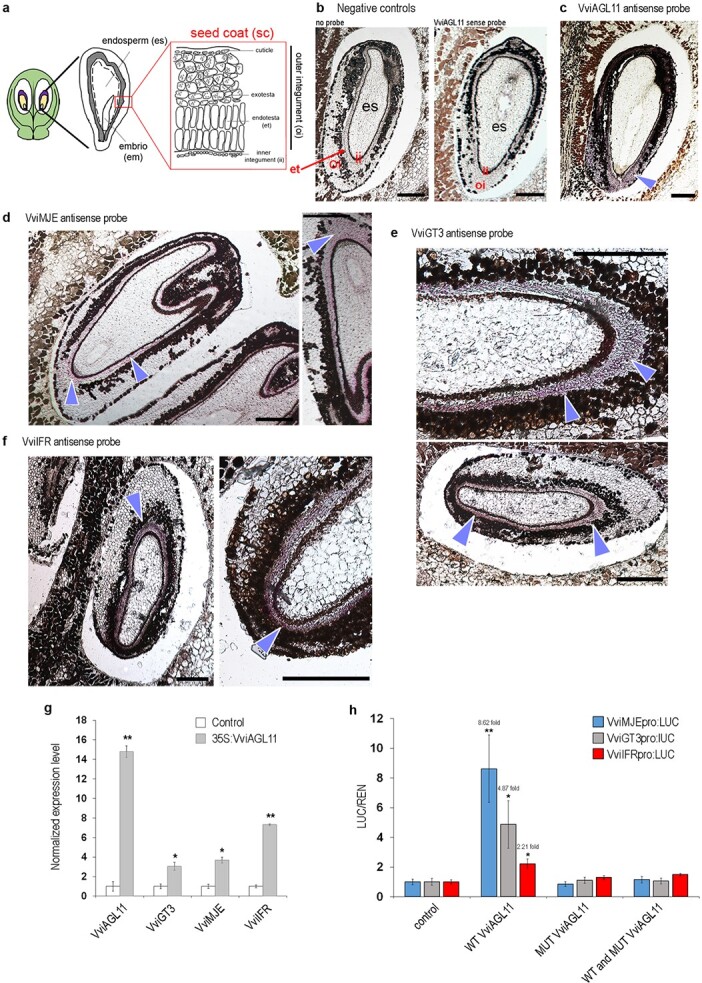
**a** Scheme of the longitudinal section of a whole berry and seed in which the VviAGL11 and candidate target transcripts were localized. The seed coat structure is highlighted. **b** Representative images of hybridizations used as negative controls for background noise determination either with no probe (left panel) or with VviAGL11 sense probe (right panel). **c-f** Tissue-specific localization of transcripts of *VvAGL11* (**c**) *VviMJE* (**d**), *VviGT3* (**e**), and *VviIFR* (**f**): the ISH signal in the endotesta layer of the seed coat is evidenced by purple staining and indicated by blue arrowheads. Em, embryo; es, endosperm; et, endotesta layer; ii, inner integument; oi, outer integument; sc, seed coat. Bars = 50 μm. **g** RT-qPCR of *VviGT3, VviMJE, and VviIFR* genes in Thompson Seedless grapevine plantlets overexpressing *VviAGL11*. Y-axis reports normalized gene expression values and induction fold changes in response to *VviAGL11* ectopic expression. RT-qPCR was performed as previously described [20]. Each value corresponds to mean ± SE of three biological replicates relative to the *VvUBIQUITIN1* internal control. Asterisks (^*^, p < 0.05; ^**^p < 0.01) indicate significant differences when compared with the control line agroinfected with an empty vector. SE, standard deviation. **h** Transactivation of *VviMJE*, *VviGT3*, and *VviIFR* regulative regions was tested by performing a Dual-Luciferase Reporter assay in infiltrated *Nicotiana benthamiana* leaves. The single and combined activity of *VviAGL11* WT and MUT CDS was tested. LUC expression values relative to REN expression and normalized against controls are reported. Negative controls were performed by infiltrating *Nicotiana benthamiana* plants with only reference vectors. Each test was performed in biological triplicate and each value was measured in triplicate. Each graphed value corresponds to mean ± SE. Asterisks (^*^, p < 0.05; ^**^p < 0.01) indicate significant

Transcript accumulation of *VviAGL11* and candidate target genes (*VviMJE*, *VviGT3,* and *VviIFR*) were firstly investigated by *in situ* hybridizations (ISH) in a whole berry at pre-veraison stage (Fig. 5a-f).

According to previous results [21], *VviAGL11* transcripts were localized in the outer integument of the seed coat (Fig. 5c). The transcripts of *VviMJE*, *VviGT3,* and *VviIFR* were specifically localized in the endotesta layer (et) of the outer seed coat integument, displaying a clear overlapping domain of expression with *VviAGL11* (Fig. 5d-f). The development of the endotesta layer of the outer seed coat integument directs further development of the seed coat, endosperm, and, consequently, embryo. These results support the theory that the involvement of all tested genes in seed development is likely controlled by *VviAGL11*.

The ability of VviAGL11 to participate in regulating expression of three selected candidate target genes was investigated *in vivo*. We infiltrated Thompson Seedless grapevine plantlets with *Agrobacterium tumefaciens* carrying the *35S:VviAGL11* (WT) overexpression construct and in the most overexpressing line the expression level of *VviMJE, VviGT3,* and *VviIFR* in comparison to the control was determined. We revealed that *VviAGL11* overexpression significantly induced *VviMJE*, *VviGT3,* and *VviIFR* expression in Thompson Seedless leaves (Fig. 5g; Figure S6).

By performing a Dual-Luciferase Reporter assay in agroinfiltrated *Nicotiana benthamiana* leaves, we also demonstrated transcriptional activity of *VviAGL1*1(WT) on regulative regions of *VvMJE, VvGT3,* and *VvIFR* (Fig. 5h). For all selected candidate genes, we isolated approximately 1.5–2.0 kb sequence upstream of the start codon and firstly assessed in cloned sequences the presence of CArG boxes (Figure S7). We found that three regulative regions tested were significantly activated by the WT variant of VviAGL11, with the *VviMJE* promoter showing stronger induction in comparison to other targets but also to VviAGL11 autoactivation (Fig. 5h, Figure S8).

We also tested the transcription activity of MUT VviAGL11, and conversely, we observed it unable to induce expression of the three candidates (Fig. 5h). Interestingly, WT VviAGL11 activity is completely inhibited by the simultaneous expression of MUT CDS, strongly confirming the dominance effect of the mutation.

## Discussion

VviAGL11 has been proved to be the major cause of seedlessness in cultivated grapevine and a causative mutation in CDS has been identified [1, 6, 11], however, its precise mechanism of action is not yet clarified.

In the present work, we investigated for the first time the whole *VviAGL11* gene sequence (promoter and CDS region) on different genotypes representing all four seedlessness classes. We defined three major alleles featuring specific promoter-CDS combinations and demonstrated that the *VviAGL11* expression level is affected by these combinations.

The low level of expression of *VviAGL11* in seedless varieties versus the seeded ones has been already reported and a *cis-*acting misexpression mechanism was proposed as a causative agent [5, 10, 21]. Other authors excluded such a possibility since they found that both mutated and wild-type alleles are expressed at a comparable level in a progeny of Red Globe x Crimson Seedless [11]. However, previously reported data are not directly comparable with those here reported, as obtained by analyzing different tissues or different developmental stages. Instead, our data, obtained on a wider genetic background on ovules and seeds at four developmental stages, revealed significant differences among VviAGL11 expression levels and among the three different alleles. Therefore, we could assume that *cis*-acting elements have a role in regulating the three identified alleles We indeed demonstrated the ability of VviAGL11 to ectopically induce its own expression, and that this ability is strictly dependent on haplotype combinations between the regulatory region and CDS, despite a possible influence of other genomic regions present in different cultivars. Moreover, although our germplasm does not include all possible combinations, a correlation was observed between the haplotype assortment, the *VviAGL11* expression level, and the seedlessness class.

VviAGL11 is a MIKC^c^-type gene, classified as a class D MADS-box gene, whose ability to establish higher-order complexes has already been demonstrated often [22], mediated by the presence of the SEP clade proteins [23]. For example, it has been reported that the grape MADS-box family transcription factor complexes VviAG2/VviSEP3 and VviAGL11/VviSEP3 form tetramers that may be involved in the formation of ovules before flowering and bloom [24]. On the other hand, we focused on the role of VviAGL11 on stenospermocarpy, studying a different developmental phase and this will likely involve different interactions. Despite not testing VviAGL11 direct interaction with other TFs, the CDS mutation was already described as located in the C-terminus protein region and thus putatively affecting the ability to establish higher-order complexes [11]. The novelty of our work was discovering the self-activation mechanism of VviAGL11 which has never been described previously.

**Figure 6:**
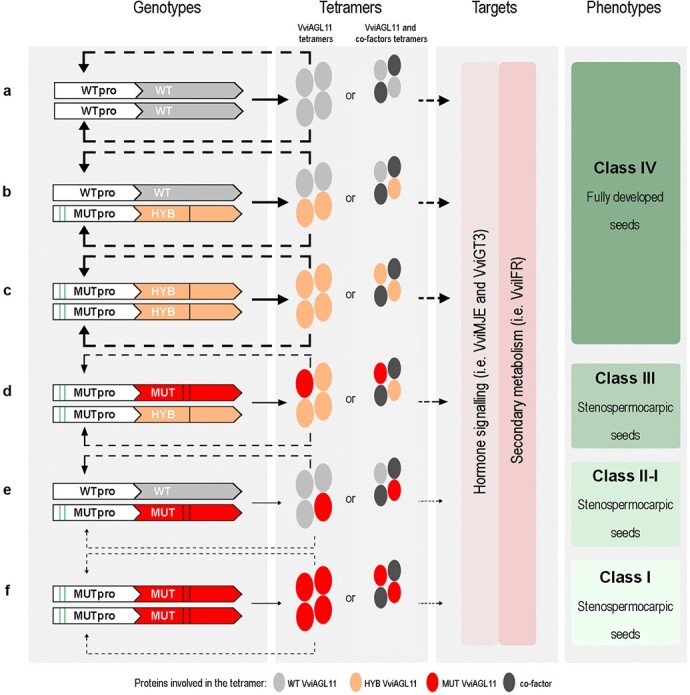
Proposed mechanism model of VviAGL11 causative effect on stenospermocarpy. Effects of haplotype combinations of promoters and CDS regions (**a**-**f**) on self-regulation of VviAGL11 transcription and regulation of target genes leading to different phenotypes. Solid lines indicate protein expression and dashed lines indicate gene regulation. Line thickness is proportional to the intensity of action simplified to three levels (i.e. high, medium and low).

These findings led us to propose a mechanism of self-activation and target activation exerted by VviAGL11 (Fig. 6).

We hypothesize that in the homozygous WT-WT CDS condition (i.e. Red Globe) the VviAGL11 protein is assembled as homo-tetramers that bind the *WTpro* and self-activate the WT CDS expression. These homo-tetramers can activate target genes allowing full seed development (Fig. 6a). In the WT-HYB CDS heterozygous condition, VviAGL11 could form hetero-tetramers able to activate both *WTpro* and *MUTpro,* to a similar extent, as observed in Pizzutello Bianco (Fig. 1c). Given that WT-HYB heterozygous cultivars are characterized by fully developed seeds (Fig. 1b), we propose that the WT-HYB hetero-tetramers can induce the expression of candidate targets similarly to the WT homo-tetramers (Fig. 6b). GUS fluorimetric assay and quantitative real-time analyses indicate that the MUT CDS has a reduced ability to self-activate its expression in comparison to that of HYB (Fig. 1c; Fig. 2). We thus propose that in the MUT-HYB CDS heterozygous condition, the HYB form prevails in the hetero-tetramers. Considering that the Afrodita cultivar, representing the MUT-HYB CDS heterozygous condition, belongs to class III of stenospermocarpy with large green vital rudiments (Fig. 1b), we propose that MUT-HYB complexes maintain the ability, albeit reduced, to activate candidate targets (Fig. 6c). In this regard, the direct activity of HYB CDS on target promoters could be further investigated. Conversely, in the WT-MUT CDS heterozygous condition (i.e. Thompson Seedless) the hetero-tetramers have reduced or no ability to self-activate their own expression and to activate putative targets, leading to a stronger stenospermocarpy phenotype (class I or II) (Fig. 6d). In our model, we speculate on a tetrameric organization of the complex involving only VviAGL11, but it should be taken into account the possibility of VviAGL11 interacting with other co-factors as previously proposed [11, 24]. As shown in Figure 6, in this latter hypothesis the combination of the WT AGL11 with the HYB or MUT protein may affect its ability to organize higher order complex and to exert its function on target genes.

Although not present in our germplasm, we included in our model the MUT-MUT and HYB-HYB CDS homozygous conditions in agreement with genotypes and phenotypes reported in previously published data. The MUT-MUT CDS homozygous condition is quite rare since *in vitro* embryo-rescue is required to obtain such crossings and, to our knowledge, only data on the whole berry are available [5] which reported very low levels of VviAGL11 transcription and extremely small seeds and berry phenotypes were recorded for all homozygous analyzed individuals. On the other hand, evidence of a seeded phenotype in an HYB-HYB CDS homozygous condition could be inferred in the variety Muskat zhemchhuzhnyi [25].

Knowing that different combinations of CDSs and regulatory regions could exist, especially in other non-vinifera species belonging to the *Vitis* genus, further studies are required to verify and improve our model. Moreover, a deeper investigation will be necessary to identify other players involved in the regulation of VviAGL11 or other partners able to form heteromeric interaction during seed coat development.

To identify candidate VviAGL11 targets, we compared the transcriptome of developing seeds, from pre-bloom to veraison, in two seeded and two seedless varieties. We highlighted an extensive difference in gene regulation between seeded and seedless varieties at the pre-veraison stage (S3), which is the stage in which *VviAGL11* shows its highest expression level in fully developed seeds. A significant upregulation in seeded varieties of genes involved in embryo development, cell growth and death, transport, secondary metabolism, and hormone signaling was revealed, corroborating previous results [25] [11]. Moreover, given the specificity of our transcriptomic experimental plan, it was possible to monitor the expression pattern of *VviAGL11* and the other identified DEGs, over ovule and seed development. This approach allowed us to achieve a wide comprehensive picture of the central role of VviAGL11 in seedlessness. STEM and *in silico* co-expression analyses highlighted that genes belonging to hormone signaling and phenylpropanoid metabolism share the same expression profile of *VviAGL11* in seeded varieties, suggesting that *VviAGL11* directly controls their expression. Interestingly, genes belonging to these functional categories were also identified in putative minor QTLs related to stenospermocarpy in grapevine [7, 26]. Despite our model being concentrated on the role of VviAGL11as positive regulator, it is known that in Arabodopsis the ortholog gene, SEEDSTICK, represses the expression of seed coat regulators, as reported also in other species [9, 27, 28]. Indeed, among the DEGs identified in the present work, the STEM analysis revealed a cluster of DEGs showing an expression profile opposite to that of VviAGL11, which showed a particularly strong down regulation in S3 when the expression level of VviAGL11 is highest. In detail, cluster 6 might include additional candidate genes negatively regulated by VviAGL11. Further studies are needed to better understand how the self-regulation mechanism can influence the negative modulator role of AGL11 on other targets.

Here we demonstrate the sole *VviAGL11* WT variant’s ability to induce expression of *GT3* and *MJE*, both involved in hormone signaling, and *IFR*, involved in secondary metabolism. These data suggest that the seedless phenotype is not only determined by the peculiar *VviAGL11* expression levels but also by the impaired target genes activation when the mutated VviAGL11 is present.

The *VviGT3* acts in hormonal homeostasis as it is involved in the synthesis of auxin-ester conjugates, fundamental for storage, transport, and reuse of auxin [29]. Auxin is considered a key component in seed development, however, the regulatory mechanisms underlying auxin synthesis and accumulation is not fully described. It was shown that auxin is produced in the endosperm after fertilization and then exported to the integuments where it triggers seed coat development by activation of downstream signaling events [30]. A general upregulation of genes involved in auxin synthesis, and a sharp increase in auxin level, were previously found in seedless berries [31]. Significant downregulation of key factors acting on the pathways controlling auxin homeostasis, such as *GH3s*, a *DIOXYGENASE FORAUXINOXIDATION-like,* and 2 *indole-3-acetate beta-glucosyltransferase*, was found in the defective endosperm 18 (de18) mutant of maize [32], and it was also recently shown that a rice indole-3-acetate beta-glucosyltransferase (OsIAGLU) regulates seed vigor possibly influencing seed development and seed germination in rice [32]. We, therefore, suggest that VviAGL11 could control the spatial distribution of the active auxin form, by inducing *VviGT3* during grapevine seed development*.*

The *VviMJE* gene is involved in demethylation of methyl jasmonate (MeJA) ester conjugates, the inactive or storage form of jasmonic acid (JA) [33]. In strawberry, it was observed that MeJA treatment affects fruit firmness altering the regulation of some cell wall metabolism-related genes and lignin accumulation [34]. In grapevine leaf, JAs induce senescence and affect the metabolism of cell wall polysaccharides [35, 36]. These observations suggest a role of *VviMJE* in the progressive thickening of the cell wall of the integumental cell layers which incorporate a large amount of pectic polysaccharides during seed coat development [36, 37]. Interestingly, it was also demonstrated that the methylesterase family catalyzes the hydrolysis of a C-O ester linkage of methyl esters not only of jasmonic acid but also of indole-3-acetic acid [38], proposing a role of these enzymes also in auxin homeostasis. Consistently, among the top list of putative VviAGL11 targets, we found the auxin-dependent protein arcA, now recognized as RACK1 (Receptor for Activated C Kinase 1), a scaffold protein that mediates hormonal responses and has a regulatory role in multiple developmental processes [39]. Altogether this evidence strengthens the hypothesis that VviAGL11 directly controls the optimal active auxin level during seed coat development.

It is well known that during seed development several flavonoids are accumulated in the innermost layer of the seed coat, playing a role in seed coat and endosperm crosstalk [40]. Interestingly, several genes involved in flavonoid metabolism have been identified as putative direct or indirect VviAGL11 targets. In detail, two *phenylalanine ammonia-lyases*, *chalcone synthase 2*, two *chalcone isomerases* (*VviCHI1* and *VviCHI2*), a *flavanone-3-hydroxylase* (F3H2), *leucoanthocyanin dioxygenase VviLDOX*, *anthocyanin reductase VviANR*, a *flavanol synthase*, and six *isoflavone reductases* have been found in the top-100 list of candidate *VviAGL11* target genes. Almost all these, flavonoid-related genes clusterized together and show a peak of expression at the S3 stage in the two seeded varieties (Fig. 4), suggesting that *VviAGL11* takes part in the control of the synthesis and accumulation of flavonoid compounds during seed development. We demonstrated that VviAGL11 directly activates one of the identified isoflavone reductases (IFRs). IFRs are unique to the plant kingdom and are considered to have crucial roles in plant response to various biotic and abiotic environmental stresses. Isoflavonoids are not abundant compounds in grapevine seeds. However, observations in soybean concerning the increase of isoflavone production in cell cultures after MeJA treatment [41] and PvIFR1’s involvement in plant growth, root development, and symbiosis, all processes in which auxin transport is involved [42], allowed us to hypothesize crosstalk among MeJA, auxin, and isoflavonoids synthesis and that this crosstalk could be orchestrated by VviAGL11. The exact functions of IFRs in grapevine remain to be elucidated.

Interestingly, VviAGL11 levels rising in the outer integument, virtually without limit, is an acceptable feature in a tissue intended to store large quantities of toxic secondary metabolites until full lignification occurs as the seed coat.

## Conclusions

In this work, we propose new insights about the VviAGL11 central role in seedlessness by identifying three target genes positively regulated by VviAGL11 and showing a dominant-negative effect of the MUT CDS on their activation. Overall, data allowed us to hypothesize a VviAGL11 mechanism of action based on networks of interactions between the different allelic products of VviAGL11 and its regulatory region. We outline a simple framework supported by functional experimental results to reasonably explain the molecular bases of partial dominance of the stenospermocarpic allele over the seeded allele. Although not conclusive, and not fully demonstrated at the protein level, this mechanism also allows us to explain the effects on fruit size through interaction with key components of hormonal signaling, which will allow the development of better breeding strategies to obtain new seedless varieties.

## Materials and methods

### Plant material

Seeded (Red Globe, Italia, Incrocio Conegliano 218, Duca di Magenta, Incrocio Pirovano 77, Incrocio Prosperi 105, Almeria and Pizzutello Bianco) and seedless (Autumn Royal, Thompson Seedless, Supernova, Afrodita) table grapevine varieties were selected from a grape collection grown in the experimental field of the Research Centre Viticulture and Enology in Turi, Italy, under conventional vine management and without hormone treatments in the 2013–2014 seasons.

*Nicotiana benthamiana* plants for transactivation assays were grown from seeds in a greenhouse with temperature between 21°C and 30°C, relative humidity of approximately 32–50% and a 15 h/9 h light/dark cycle.

Thompson Seedless plantlets for VviAGL11 transient overexpression were *in vitro* micro-propagated and cultivated in a growth chamber at 25°C with a 16-h photoperiod.

### Isolation and cloning

By using the Phusion® High-Fidelity DNA Polymerase (Biolabs), the CDS of *VviAGL11* was isolated from Italia, Thompson Seedless, and Afrodita cDNAs by extracting total RNA from seeds at pea size and performing retrotranscription with oligo dT. The regulative region of *VviAGL11* (2147 bp) was isolated from Italia and Afrodita genomic DNA, and the regulative region of *VviMJE* (1145 bp), *VviGT3* (922 bp) and *VviIFR* (1427 bp) was amplified from Italia genomic DNA. Genomic DNA was extracted from leaves with DNeasy Plant mini-column kit (Qiagen, Hilden, Germany).

The isolated sequences were directionally cloned into the Gateway entry vector pENTR/D-TOPO (Invitrogen, Waltham, MA, USA) and transferred by site-specific LR recombination into a specific binary vector (http://www.vib.be/en/research/services/Pages/Gateway-Services.aspx). The *VviAGL11* CDSs were transferred into the pK7GW2,0 overexpression vector. The *VviAGL11* regulative regions were transferred into the pKGWFS7 reporter vector to control *GFP* and *GUS* expression. The *VviMJE*, *VviGT3, VviIFR*, and the WT *VviAGL11* regulative regions were transferred into the pPGWL7.0 reporter vector to control the *Firefly Luciferase* gene (*LUC*) expression. All cloned sequences have been checked with Sanger sequencing.

### *VviAGL11* gene sequencing

*VviAGL11* gene sequences (promoter and CDS) were amplified from genomic DNA of table grapevine varieties by using the Phusion Hot Start II DNA Polymerase (Thermo Fisher Scientific). PCR products were purified from agarose gel using Illustra GFX PCR DNA and Gel Band Purification Kit (Merck KGaA, Darmstadt, Germany) and then sequenced by Ion Torrent technology (Thermo Fisher Scientific). Amplicon libraries were prepared by using the Ion Plus Fragment Library Kit (Thermo Fisher Scientific) following manufacturer instructions. The enriched library was loaded on Ion PGM 314 chip and sequenced on the Ion PGM™ sequencer using Ion PGM™ Sequencing 200 Kit v2. Reads were aligned on the grapevine reference genome (12X.v2) of Pinot Noir [43]. The depth of sequencing obtained was approximately 800X. Variant caller plugin provided in the Torrent Suite Software was run for the identification of polymorphisms across the reference. Sequences were viewed by the Integrative Genomics Viewer (IGV) [44].

### GUS Fluorimetric assay

The pK7WG2.0 vectors containing the *VviAGL11* CDSs and the pKGWFS7 vectors harboring the *VviAGL11* regulative regions were transferred to *A. tumefaciens* EHA105 strain by electroporation. A GUS Fluorimetric Assay was performed in *Nicotiana benthamiana* leaves infiltrated as previously described [45]. Frozen leaves collected 72 h after *Agrobacterium*-mediated infection were homogenized in Extraction Buffer (NaHPO_4_ 1 M pH 7.0, Na_2_EDTA 0.5 M pH 8.0, Sarcosyl, Triton X-100 10%, 2-Mercaptoethanol 10 mM) and centrifuged at 13000 rpm for 2^m^. A 1:10 dilution of the extracted protein was incubated at 37°C with Assay Solution (10 mg of MUG in 10 ml of Extraction Buffer) and after 1 h the reaction was stopped with an equal volume of NaCO_3_ 0.2 M and the fluorescence (Exc. 355 nm - Em. 460 nm) was detected. To calculate the amount of MUG converted to 4-MU in 1 h, a T0 plate stopping the reaction immediately after the addition of the Assay Solution was also processed.

### Transcriptomic assays on grapevine ovules and developing seeds

Total transcriptome assays were performed on ovules and developing seeds derived from two seeded (Italia and Red Globe) and two seedless (Thompson Seedless and Autumn Royal) table grape varieties. Four developing stages were considered: pre-bloom (BBCH 57–60; E-L 17), post-bloom (BBCH 65–68; E-L 19–25), pea size (BBCH 73–75; E-L 29) and veraison (BBCH 81; E-L 35) (Lorenz, Eichhorn et al. 1995) (Fig. 3a). Ovules were extracted from pre-bloom flowers under a stereomicroscope with the aid of tweezers and needles. Upon extraction, ovules were immediately transferred in a centrifuge tube containing 50 μl of water to prevent drying and kept in ice. When enough ovules were collected (50 to 100 depending on size) tubes were spun and water was removed then snap-frozen in liquid nitrogen.

Total RNA was extracted from 0.1 g of ovules or seeds collected at each experimental stage with Total RNA Isolation Mini Kit (Agilent Technologies). RNA integrity was assessed by automated gel electrophoresis on 2100 Bioanalyzer (Agilent Technologies). The cDNA synthesis, labeling hybridization, and washing were performed according to the Agilent Microarray-Based Gene Expression Analysis Guide (6.9.1). Hybridization was carried out in an Agilent custom microarray (https://www.ncbi.nlm.nih.gov/geo/query/acc.cgi?acc=GPL26142). The array images were analyzed using the Agilent Feature Extraction software version 12.0. Microarray expression data were normalized by the 75 percentile threshold and negative signals were filtered out. Functional annotation was implemented accordingly to the V1 version of the 12X draft release of the grapevine genome and functional categories distribution [45] (Data S1). Differentially expressed genes (DEGs) were determined by performing a t-test using TMeV software (https://sourceforge.net/projects/mev-tm4/). with a p-value of 0.05%. DEGs were grouped by VENN diagrams (http://bioinformatics.psb.ugent.be/) and DEG patterns in all stages and varieties were investigated by applying the STEM clustering method [46].

### In situ hybridization (ISH)

Samples were collected from *Vitis vinifera* cv. Corvina clone 48 grown in Montorio (45° 27′ 17” North, 11° 03′ 14″ East, Verona, Italy) in a commercial vineyard in the 2010-2011growing seasons. Berries were collected 8 weeks before veraison (E-L stage 34). Tissue fixation, embedding, and sectioning were carried out as previously described [47]. ISH steps were performed as previously described [48]. *VviAGL11, VviMJE, VviGT3* and *VviIFR* probes were prepared by PCR on cloned fragments obtained from seeds cDNA of *V. vinifera* cv. Corvina [47].

### Expression analysis in grapevine leaves transiently overexpressing *VviAGL11*

Five *in vitro* grapevine Thompson Seedless plantlets were agroinfiltrated with *A. tumefaciens* C58C1 strain, either transformed with the *35S:VviAGL11wt* construct with a pK7WG2.0 control vector containing a non-coding sequence [49]. Total RNA of *Agrobacterium*-infiltrated grapevine was isolated from ~20–40 mg of young and well-expanded ground leaves using Spectrum™ Plant Total RNA kit (Merck KGaA). RNA samples were quantified with the NanoDrop 2000 instrument (Thermo Fisher Scientific) and 1 μg aliquots were treated with DNase I (Ambion, Thermo Fisher Scientific) and reverse transcribed using Super-Script™ III Reverse Transcriptase (Invitrogen). The expression level of *VviAGL11*, *VviMJE*, *VviGT3,* and *VviIFR* was determined by quantitative real-time polymerase chain reaction (RT-qPCR) in a transiently transformed grapevine. Leaves were chosen to avoid the dominant effect of the mutation on the target genes expression induction, because, according to Fasoli et al. (2012) the *VviAGL11* expression level in leaves is negligible.

### Dual-luciferase reporter assay

The pK7WG2.0 vectors containing the *VviAGL11* CDSs and the pPGWL7 vectors harboring the *VviMJE, VviGT3, VviIFR*, and the WT *VviAGL11* regulative regions were transferred to *A. tumefaciens* EHA105 strain (Hellens, Mullineaux et al. 2000) by electroporation. Dual-Luciferase Reporter Assay was performed in *Nicotiana benthamiana* leaves infiltrated as previously described [45]. The assay was performed on fresh leaf disks collected 72 h after *Agrobacterium*-mediated infection and following the manufacturer’s instruction (Promega). A reference vector overexpressing the *Renilla reniformis* luciferase gene (*REN*) was used to normalize LUC luminescence. REN and LUC luminescence were detected using a Tecan Infinite ® M200 PLEX instrument.

### Bioinformatics

Co-expression analysis based on Pearson’s correlation coefficient was carried out using Cor.To tool and *VviAGL11* (*VIT_18s0041g01880*) as bait in the entire transcriptome of ovules and developing seeds and in the published Corvina atlas [18] (Data S6). Correlation analysis of *VviAGL11* expression was also performed exploiting the grapevine VTCdb database (http://vtcdb.adelaide.edu.au/Home.aspx) selecting HRR co-expression measure [19]. All the genes resulted by the co-expression analysis were sorted by assigning a value to a) concordance between the different data sources, b) intensity of correlation with *VviAGL11* given by multiplying each value of correlation from the different data source, c) levels of expression in the target tissues according to the overall intensity of fluorescence determined in our transcriptomic analysis. A final score to each gene was given by multiplying a)^*^b)^*^c) and finally all genes in the dataset were sorted accordingly from highest to lowest (Data S6). A minimum averaged expression level in ovules and seed samples was also considered as a positive parameter and this combined with parameter b) allowed us to focus our attention on those genes positively correlated to *VviAGL11* which indeed are putatively activated by VviAGL11, in order to make easier their functional validation.

All the primer sets used are listed in Table S2.

### Accession numbers

*VviAGL11* data sequences reported in this paper have been deposited in the Sequence Read Archive (SRA) of NCBI under the BioProject accession number PRJNA816322 (Biosample accession numbers from SAMN26669120 to SAMN26669128).

Microarray data of the transcriptome of the ovule and seed samples are available at GEO under the series entry GSE186838 (https://www.ncbi.nlm.nih.gov/geo/query/acc.cgi?acc=GSE186838).

## Supplementary Material

Web_Material_uhac133

## Data Availability

The authors confirm that the data supporting the findings of this study are available within the article and its supplementary material, at GEO for the Microarray data, and at NCBI SRA for sequences data.
