# Supplementary material for: VviAGL11 self-regulates and targets hormone- and secondary metabolism-related genes during seed development
Source: Hortic Res. 2022 Jun 10;9:uhac133. doi: 10.1093/hr/uhac133 (PMC9433981; doi:10.1093/hr/uhac133)
Supplement: Web_Material_uhac133 [file web_material_uhac133.zip › Web_Material_uhac133/Supplemental Data_clean.docx]

**Supplemental Data**

**VviAGL11 self-regulates and targets hormone and secondary metabolism crosstalk during seed development**

Alessandra Amato, Maria Francesca Cardone, Nallatt Ocarez, Fiammetta Alagna, Benedetto Ruperti, Chiara Fattorini, Riccardo Velasco, Nilo Mejía, Sara Zenoni^*^, Carlo Bergamini^*^

**Figure S1.** Assessment of the allelic specificity of primer pairs reported in Table S2.

**Figure S2.** *VviAGL11* regulatory regions clustering analysis.

**Figure S3.** WT *VviAGL11* promoter region analyses.

**Figure S4.** Venn diagram of common and specific differentially expressed genes (DEGs) between SD and SL varieties at each developmental stage.

**Figure S5.** Clustering analysis of the 5971 DEGs identified at S3 stage (SDvsSL and/or RGvsTS) during the entire ovule and seed development in the four varieties by STEM approach.

**Figure S6.** *VviAGL11* expression level in agroinfiltrated grapevine Thompson Seedless plantlets.

**Figure S7.** CArG boxes identification in the *VviAGL11* candidate target genes promoter region.

**Figure S8.** Validation of the WT *VviAGL11* self-induction.

**Table S1.** Variant caller output for the Ion PGM™ sequencing of VviAGL11 whole gene in nine table grape varieties.

**Table S2.** List of primers used.

**Data S1.** Ovules and developing seeds transcriptome of two seeded (Italia and Red globe) and two seedless (Thompson Seedless and Autumn Royal) table grape varieties.

**Data S2.** Differentially expressed genes (DEGs; p<0.05; |FC|>2) between SD and SL varieties at each developmental stage.

**Data S3.** Differentially expressed genes (DEGs; p<0.05; |FC|>2) between RG and TS varieties at stage S3.

**Data S4.** Commonly up and downregulated genes among differentially expressed genes (DEGs; p<0.05; |FC|>2) identified at stage S3 in both the SDvsSL and RGvsTS comparisons.

**Data S5.** STEM analysis on the four varieties developing seed datasets.

**Data S6.** Multi-*VviAGL11* co-expression analysis.

**Supplemental Figures**

**
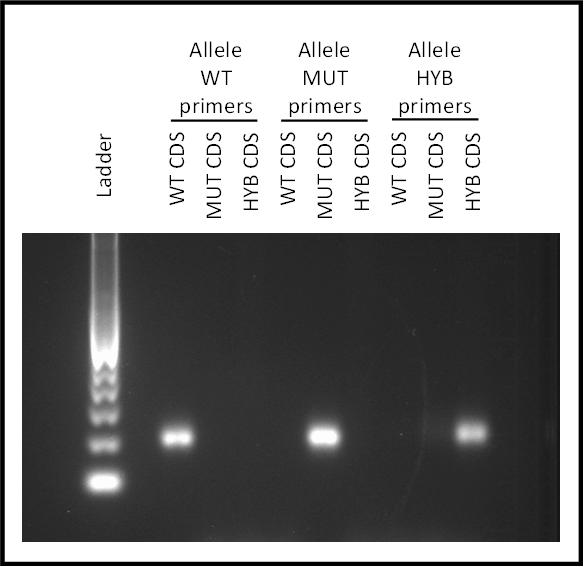
**

**Figure S1.** Assessment of the allelic specificity of primer pairs reported in Table S2. Primers were used in Quantitative real-time polymerase chain reaction (RT-qPCR) described in Figure 1C. PCR conditions: 30 cycles, 50 ng of template WT, MUT and HYB cloned CDSs, 60°C annealing temperature.

**
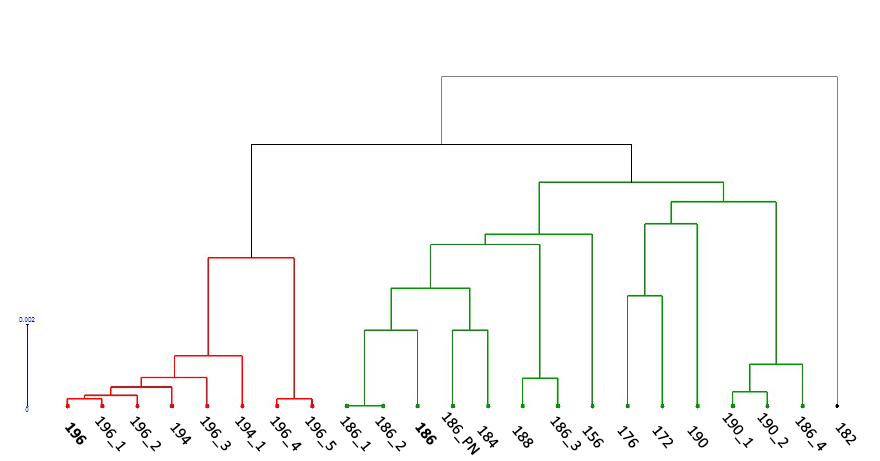
**

**Figure S2. *VviAGL11* regulatory regions clustering analysis**. UPGMA Hierarchical Clustering was applied to the upstream region of *VviAGL11* (-2993 to -1 bp from transcriptional starting site). Dissimilarity was calculated on Sanger sequencing data previously obtained^1^ using DARwin software (available online at <http://darwin.cirad.fr/darwin> Retrieve on 11^th^ July, 2014). Default settings were used for dissimilarity of sequence data (Missing Data gestion: Pairwise site deletion and 90% of minimal proportion of valid data; Gaps gestion: pairwise gap block correction and minimal size set to 1) and also for Hierarchical Clustering (unweighted pair group method using average UPGMA). Promoters are named according to their p3_VviAGL11 SSR size. PN40024 reference genome promoter is 186_PN. MUTpro and WTpro regulatory regions used for all experiments performed in this paper are in bold and named respectively 196 and 186. Promoters are clustered in two major groups (coloured in red for MUTpro and green for WTpro) and one outlier (182).

**
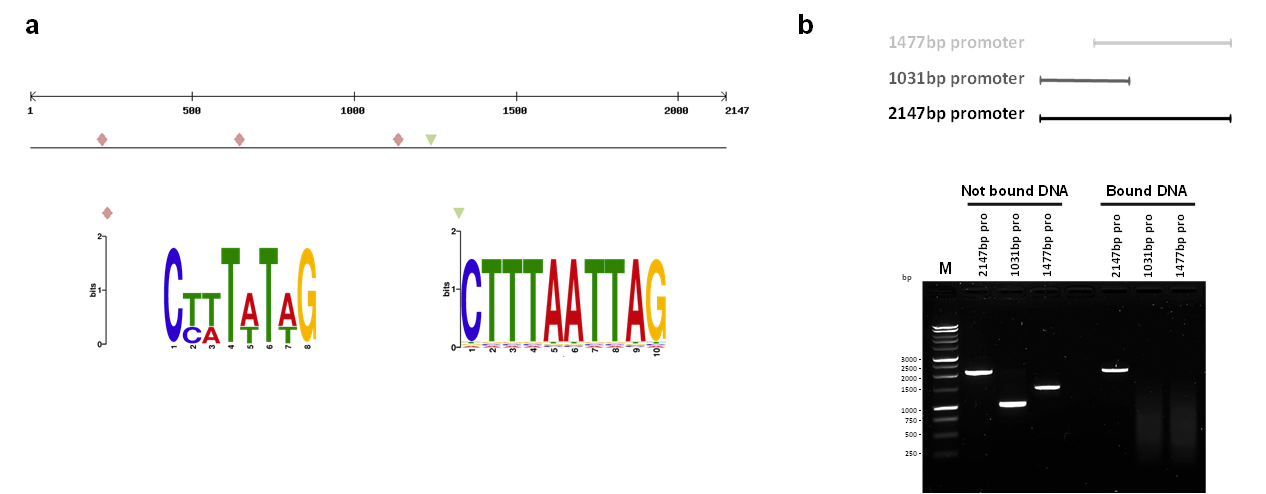
**

**Figure S3. WT *VviAGL11* promoter region analyses.** **a** CArG boxes identification in the WT VviAGL11 regulative region. The isolated and cloned DNA sequence was screened for *cis*-acting elements by using the The Plant Promoter Analysis Navigator (PlantPAN; http://PlantPAN2.itps.ncku.edu.tw) and CArG boxes were identified by searching for the CC(A/T)_6-8_GG consensus sequence^2,3^. The motif comparison of the same length DNA binding sites (represented by the same position indicator) was retrieved by using the Tomtom tool (https://meme-suite.org/meme/tools/tomtom). **b** Preliminary protein-DNA affinity assay. The affinity of the WT VviAGL11 protein with the WT *VviAGL11* regulative region (2147bp) and with two fragments of it (a 1031bp sequence and a 1477bp sequence schematized on the top) was preliminary tested. The WT *VviAGL11* sequence cloned in the pENTR/D‐TOPO was transferred to the Gateway destination vector pIX‐HALO^4^. The HALO‐VviAGL11 fusion protein was *in vitro* translated using the TNTR SP6 coupled reticulocyte lysate system (Promega). Separate aliquots of the obtained protein were immobilized to the Magne® HaloTag® Beads (Promega) and mixed with 150 ng of DNA. Protein-DNA incubation and bound DNA elution were performed according to the DAP‐seq protocol described by Galli et al.^5^. Putative bound DNA sequences were amplified using primer sets listed in Table S2 and gel visualized. Not bound DNAs were also run and we observed that only the 2147 bp *VviAGL11* promoter is detectable before and after the affinity assay suggesting that it is likely bound by the VviAGL11 protein.

**
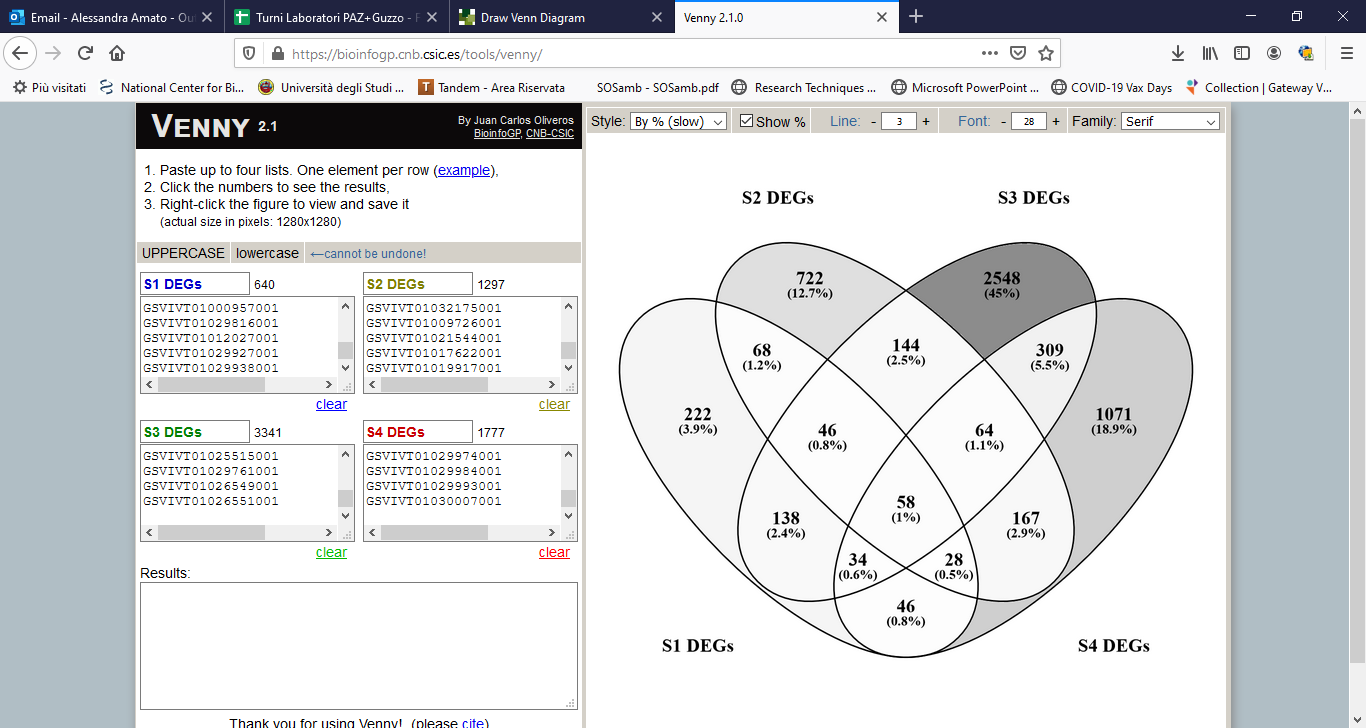
**

**Figure S4. Venn diagram of common and specific differentially expressed genes (DEGs) between SD and SL varieties at each developmental stage.**

**
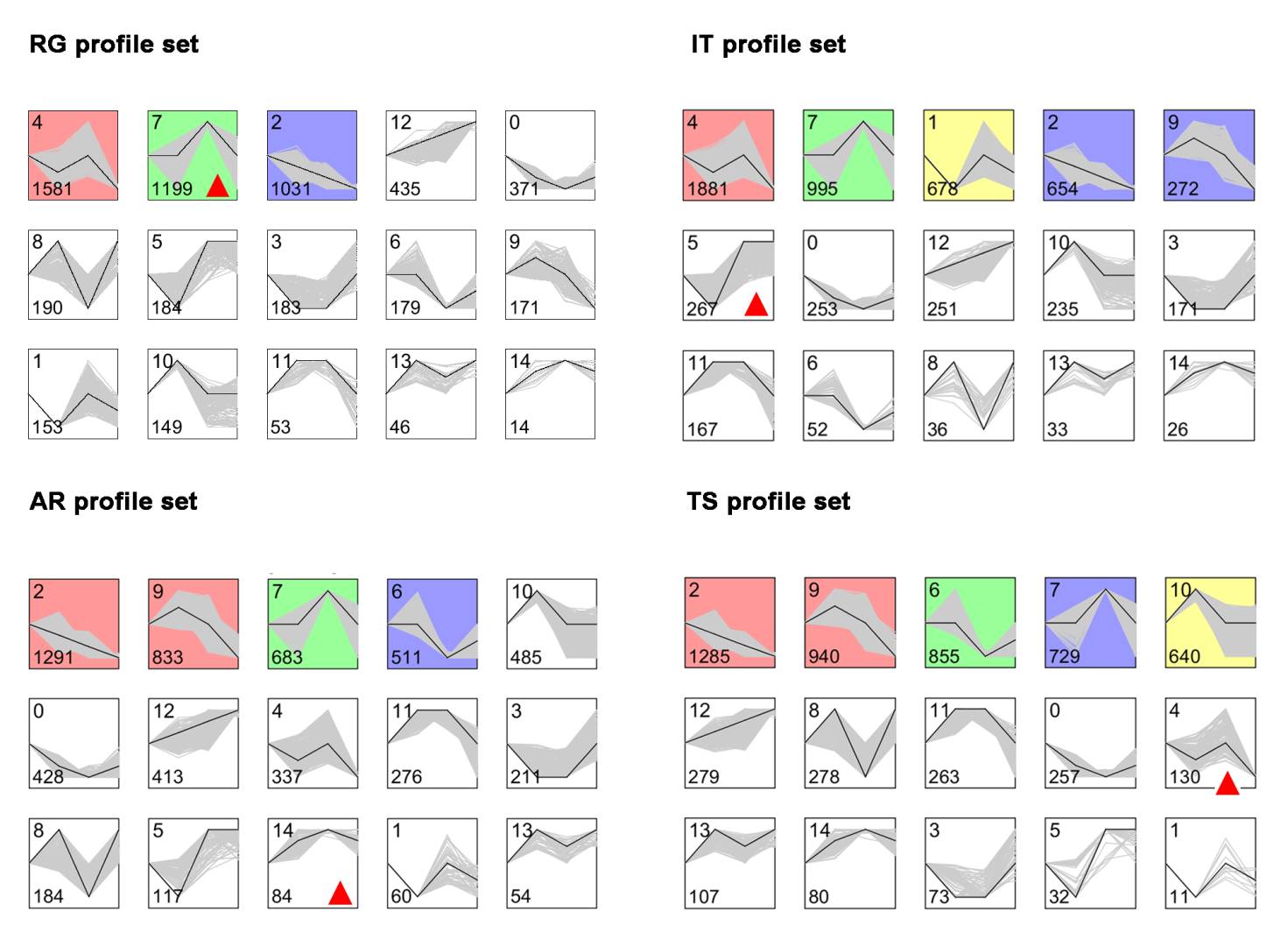
**

**Figure S5. Clustering analysis of the 5971 DEGs identified at S3 stage (SDvsSL and/or RGvsTS) during the entire ovule and seed development in the four varieties by STEM approach.** In the 15 frames for each variety, the number of genes is displayed to the bottom left, the number ID is shown to the top left, g*ray curves* represent individual profiles and the *black line* represents the averaged profile. The x-axis represents sampling points and the y-axis denotes log_2_ scale fold change in expression value. The significant clusters are colored and red triangles mark the *VviAGL11* including cluster.

**
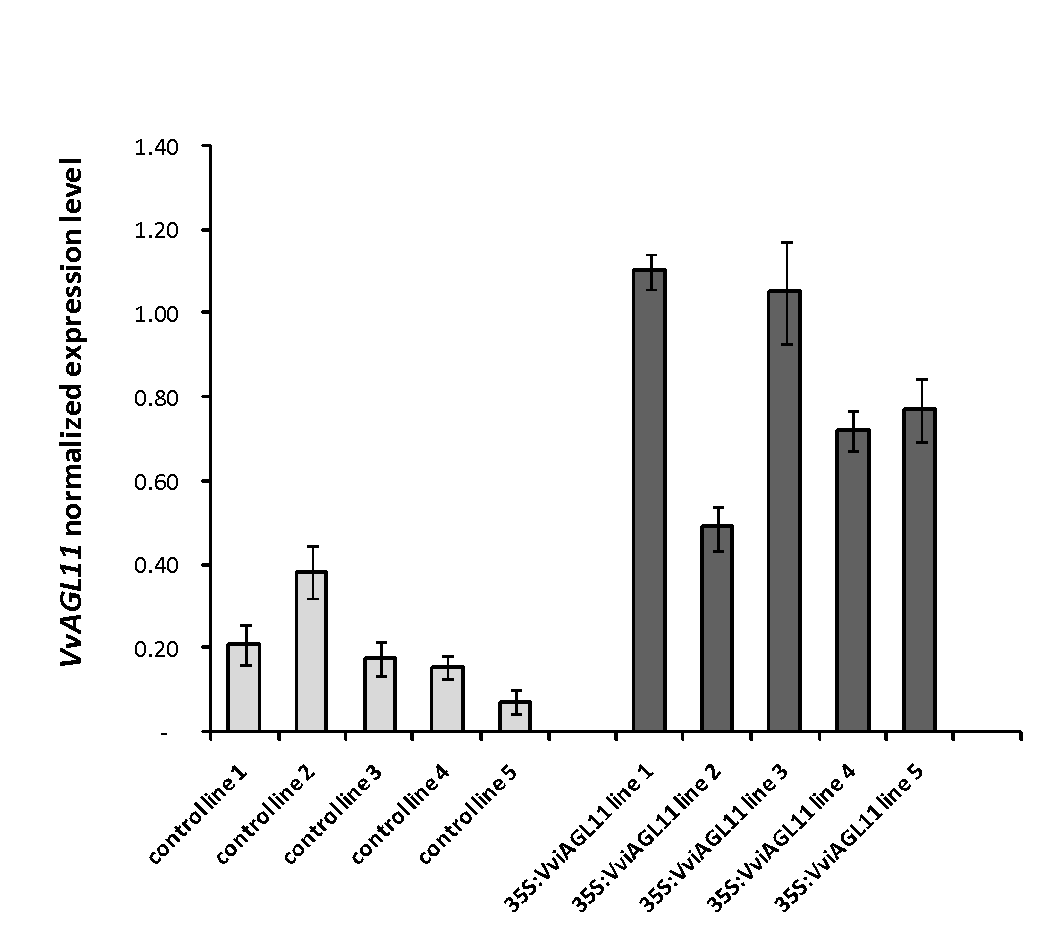
**

**Figure S6. *VviAGL11* expression level in agroinfiltrated grapevine Thompson Seedless plantlets.** *VviAGL11* expression level in control and overexpressing (35S:VviAGL11) lines was determined by RT-qPCR performed as previously described^6^. Each value corresponds to the mean ± SE of three biological replicates relative to the *VvUBIQUITIN1* internal control. Asterisks (*) indicate the control and the *VviAGL11* overexpressing lines used to further investigate the *VviMJE, VviGT3* and *VviIFR* expression level. SE, standard error.

**
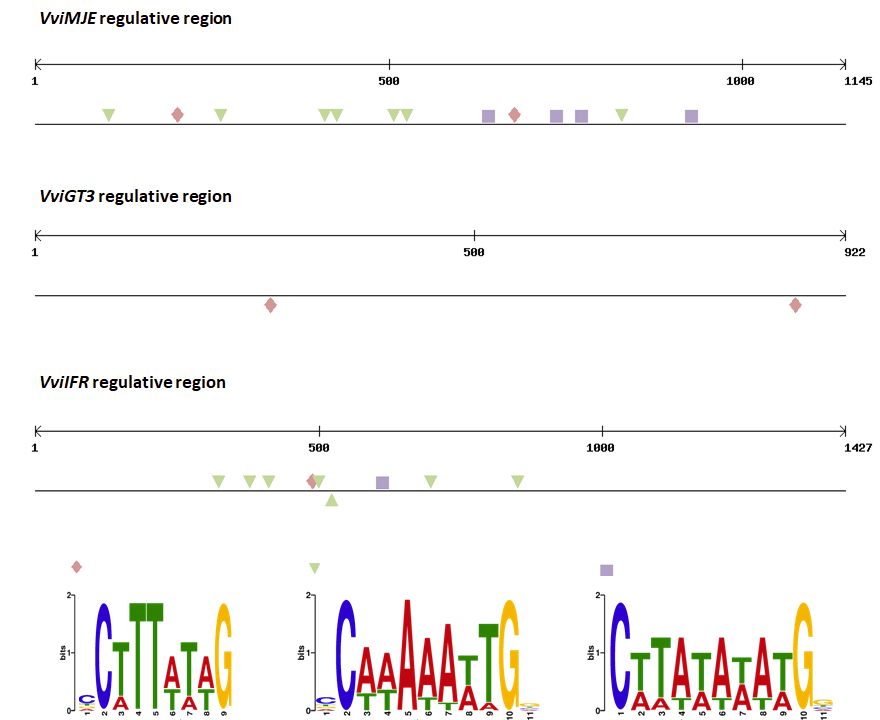
**

**Figure S7. CArG boxes identification in the VviAGL11 candidate target genes promoter region.** All the isolated, cloned and tested for VviAGL11 transactivation DNA sequences (**Figure 5c**) were screened for *cis*-acting elements by using the The Plant Promoter Analysis Navigator (PlantPAN; http://PlantPAN2.itps.ncku.edu.tw) and CArG boxes were identified by searching for the CC(A/T)_6-8_GG consensus sequence^2^. The motif comparison of the same length DNA binding sites (represented by the same position indicator) was retrieved by using the Tomtom tool (https://meme-suite.org/meme/tools/tomtom).

**
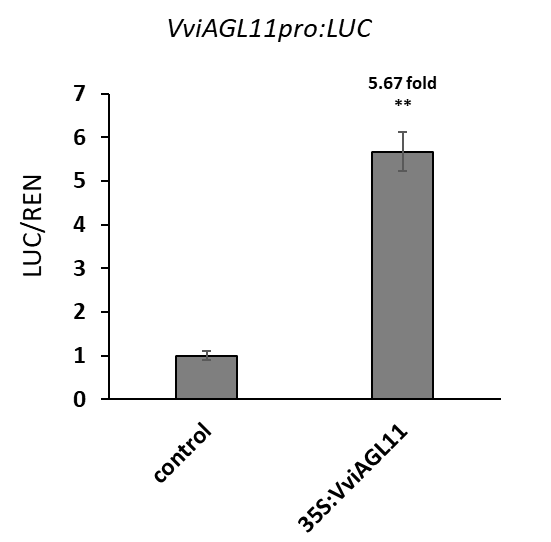
**

**Figure S8. Validation of the WT *VviAGL11* self-induction.** The capability of the VviAGL11 WT protein to activate the *WT VviAGL11* regulative region was tested by performing a Dual-Luciferase Reporter assay in inﬁltrated *Nicotiana benthamiana* leaves. LUC expression values relative to the REN expression and normalized against controls is reported. Negative control was performed by infiltrating *Nicotiana benthamiana* plants with only reference vectors. Each test was performed in biological triplicate and each value was measured in triplicate. Each graphed value corresponds to the mean ± SE. Asterisks (**p < 0.01) indicate significant differences in promoter activation in comparison to the respective negative control; SE, standard error.

**Table S1.** Variant caller output for the Ion PGM™ sequencing of *VviAGL11* whole gene in nine table grape varieties. Variants found in VviAGL11 genomic region in grape varieties (Afrodita, Almeria, Conegliano Precoce 218, Duca di Magenta, Incrocio Pirovano 77, Incrocio Prosperi, Red Globe, Regal, Supernova seedless). Sequencing was performed on amplicons, variants were found from position 30.305.666 to 30.777.932 on chromosome 18. In position 30.306.420 is highlighted the T to C variant located in exon 7 shared by both the MUT and the HYB coding sequences. In position 30.306.458 is highlighted the variant C to A specific of the MUT CDS.

**Table S2.** List of primers used. For allele-specific primers the last position (in bold) is sequence-specific, and mismatches introduced to increase the selectivity of amplification are reported (in red).


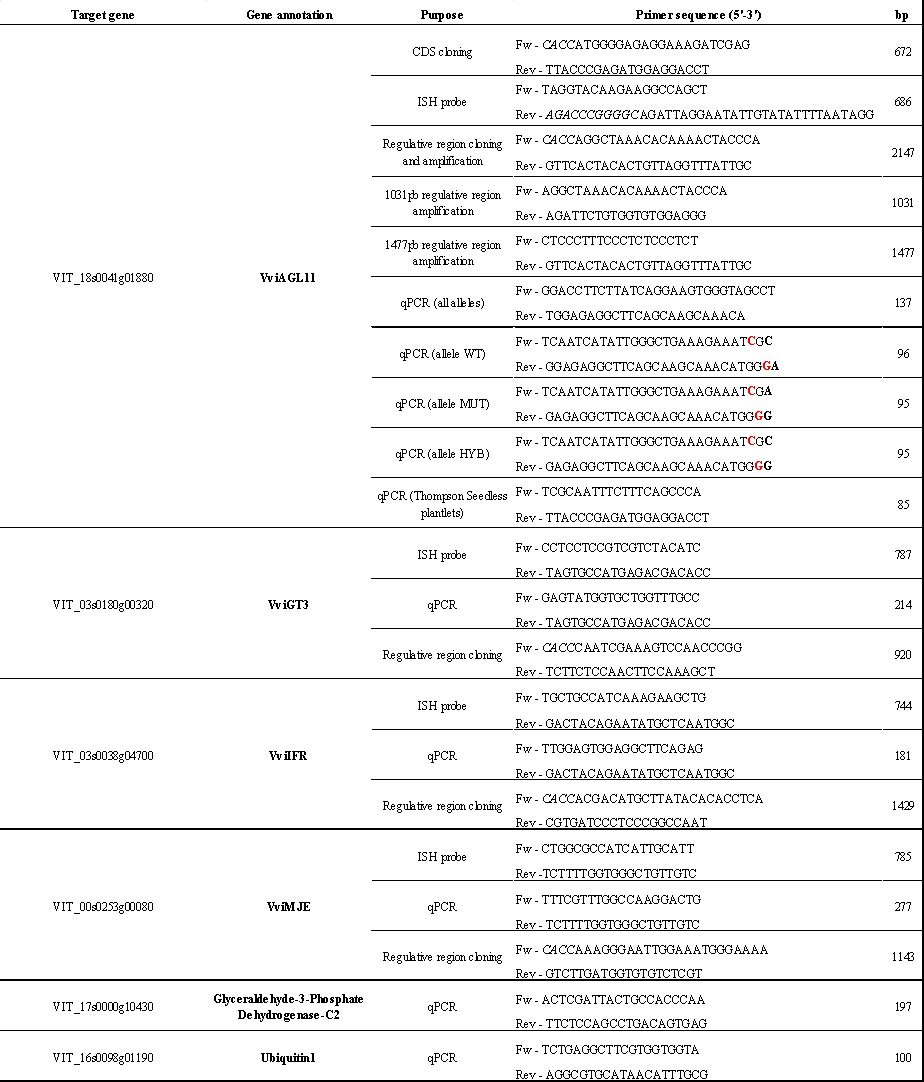


**Data S1. Ovules and developing seeds transcriptome of two seeded (Italia and Red Globe) and two seedless (Thompson Seedless and Autumn Royal) table grape varieties**. Ovules and developing seeds transcriptome of two seeded (Italia and Red Globe) and two seedless (Thompson Seedless and Autumn Royal) table grape varieties. Four developing stages were considered (S1-S4) and each sample has been tested in biological triplicate (48 samples in total). For each gene, the probe name, the gene ID, the gene functional annotation, the functional category and the fluorescence values are indicated. Functional annotation was reported accordingly to the V1 version and functional categories distribution^7^.

**Data S2. Differentially expressed genes (DEGs; p<0.05; |FC|>2) between SD and SL varieties at each developmental stage.** Differentially expressed genes (DEGs; p<0.05; |FC|>2) between SD and SL varieties at each developmental stage. For each gene, the probe name, the gene ID, the gene functional annotation, the averaged fluorescence values, and the FC (SD/SL) are indicated. Functional annotation was reported according to the V1 version of the grapevine reference genome. FC, Fold Change.

**Data S3. Differentially expressed genes (DEGs; p<0.05; |FC|>2) between RG and TS varieties at stage S3.** For each gene, the probe name, the gene ID, the gene functional annotation, the fluorescence values, and the FC (RG/TS) are indicated. Functional annotation was reported according to the V1 version of the grapevine reference genome. FC, Fold Change.

**Data S4. Commonly up and downregulated genes among differentially expressed genes (DEGs; p<0.05; |FC|>2) identified at stage S3 in both the SDvsSL and RGvsTS comparisons.** For each gene, the gene ID, the gene functional annotation, and the functional category are indicated. Functional annotation was reported accordingly to the V1 version and functional categories distribution^7^. FC, Fold Change.

**Data S5. STEM analysis on the four varieties developing seed datasets.** For each gene, the gene ID, the including cluster, and the expression value at each developing stage normalized on the S1 value set at zero by the software, are indicated.

**Data S6. Multi-*VviAGL11* co-expression analysis.** *VvAGL11* co-expression analysis was performed in the entire developing ovule and seeds transcriptome from Dataset S1, in the grapevine cv. Corvina expression atlas^8^ and in the VTC database^9^. For genes co-expressed with *VviAGL11* in at least one dataset (n=6,967), the relative Pearson Correlation Coefficient (PCC; columns D-E), the seeded/seedless ratio identified at 3 seed developmental stages by Wang *et al.^10^* and the assigned scoring, are reported. A positive PCC value refers to a positive correlation of expression with *VviAGL11*, while a negative PCC value indicates a negative (divergent or opposite) one. The final scoring was calculated in the last column as described in the pertinent result and material and methods sections. Functional annotation was reported according to the V1 version and functional categories distribution^7^.

**Supplemental reference****s**

1. Ocarez, N. *et al.* Unraveling the Deep Genetic Architecture for Seedlessness in Grapevine and the Development and Validation of a New Set of Markers for VviAGL11-Based Gene-Assisted Selection. *Genes (Basel)* **11**, <http://dx.doi.org/10.3390/genes11020151> (2020).

2. Mendes, M.A. *et al.* MADS domain transcription factors mediate short-range DNA looping that is essential for target gene expression in Arabidopsis. *Plant Cell* **25**, 2560-72 <http://dx.doi.org/10.1105/tpc.112.108688> (2013).

3. Bhupinder, S.a.R.G.F. Redundant CArG Box Cis-motif Activity Mediates SHATTERPROOF2 Transcriptional Regulation during Arabidopsis thaliana Gynoecium Development. *Frontiers in plant science* **8**, <http://dx.doi.org/https://doi.org/10.3389/fpls.2017.01712> (2017).

4. Bartlett, A. *et al.* Mapping genome-wide transcription-factor binding sites using DAP-seq. *Nat Protoc* **12**, 1659-1672 <http://dx.doi.org/10.1038/nprot.2017.055> (2017).

5. Galli, M. *et al.* The DNA binding landscape of the maize AUXIN RESPONSE FACTOR family. *Nat Commun* **9**, 4526 <http://dx.doi.org/10.1038/s41467-018-06977-6> (2018).

6. Zenoni, S. *et al.* Characterization of transcriptional complexity during berry development in Vitis vinifera using RNA-Seq. *Plant Physiol* **152**, 1787-95 <http://dx.doi.org/10.1104/pp.109.149716> (2010).

7. Grimplet, J. *et al.* Comparative analysis of grapevine whole-genome gene predictions, functional annotation, categorization and integration of the predicted gene sequences. *BMC Res Notes* **5**, 213 <http://dx.doi.org/10.1186/1756-0500-5-213> (2012).

8. Fasoli, M. *et al.* The grapevine expression atlas reveals a deep transcriptome shift driving the entire plant into a maturation program. *Plant Cell* **24**, 3489-505 <http://dx.doi.org/10.1105/tpc.112.100230> (2012).

9. Wong, D.C., Sweetman, C., Drew, D.P. & Ford, C.M. VTCdb: a gene co-expression database for the crop species Vitis vinifera (grapevine). *BMC Genomics* **14**, 882 <http://dx.doi.org/10.1186/1471-2164-14-882> (2013).

10. Wang, L. *et al.* Transcriptome analyses of seed development in grape hybrids reveals a possible mechanism influencing seed size. *BMC Genomics* **17**, 898 <http://dx.doi.org/10.1186/s12864-016-3193-1> (2016).
